# DMG-GCN: A Dynamic Microstate-Guided Graph Convolutional Network for EEG Cognitive Workload Decoding in Air Traffic Control

**DOI:** 10.3390/bios16080452

**Published:** 2026-08-20

**Authors:** Yu Zhang, Quan Shao, Haihong Yang, Xiaosong Ren, Xiaolin Peng

**Affiliations:** 1 College of Civil Aviation, Nanjing University of Aeronautics and Astronautics, Nanjing 211106, China; zhangyu0309@nuaa.edu.cn; 2 Key Laboratory of Civil Aviation Emergency Science & Technology, Nanjing University of Aeronautics and Astronautics, Nanjing 211106, China; 3 Operation Supervisory Center, Civil Aviation Administration of China, Beijing 100710, China; hh_yang1@caac.gov.cn (H.Y.); renxiaosongcaac@163.com (X.R.); 4 Guangzhou Baiyun International Airport Co., Ltd., Guangzhou 510470, China; pengxiaolin@gdairport.com

**Keywords:** cognitive workload, electroencephalography (EEG), brain–computer interface (BCI), air traffic control, graph convolutional network (GCN), microstate analysis, feature disentanglement

## Abstract

Complex inter-subject variability induces severe distribution shifts in the physiological features of electroencephalography (EEG) for air traffic controllers (ATCOs). These inter-subject shifts limit the generalization and interpretability of passive brain–computer interfaces (pBCIs) during cognitive workload decoding. To address this, a Dynamic Microstate-Guided Graph Convolutional Network (DMG-GCN) is proposed for robust cross-subject workload recognition. This approach utilizes a Dynamic Selective Kernel Temporal Convolutional Block (DSK-TCB) to adaptively extract multi-scale temporal–spectral dynamics, while concurrently constructing a time-evolving adjacency matrix via a Microstate-Guided Dynamic Graph Block (MG-DGB) to disentangle topological sub-networks. A spatiotemporal graph convolution module then aggregates these representations, and a temporal self-attention mechanism focuses on task-critical transition moments. Extensive experiments on simulated multi-level air traffic control tasks demonstrate that the proposed model achieves an overall average accuracy of 80.30% and an average F1-score of 78.63% in cross-subject evaluations, significantly outperforming state-of-the-art baselines. Moreover, an exploratory interpretability analysis suggests that the extracted topological sub-networks exhibit spatial patterns consistent with specific brain network reorganizations, which encompass the transition from global distributed monitoring to temporal multimodal integration and parietal–occipital parallel processing during workload regulation. The framework provides a robust and analytically transparent pBCI solution for adaptive automation in modern aviation.

## 1. Introduction

Air traffic controllers (ATCOs) are the core decision-makers in the air transportation system, maintaining safe and efficient separation by continuously monitoring aircraft dynamics and issuing critical clearances [1,2]. Operating in a quintessential high-risk and highly dynamic environment, ATCOs are required to sustain complex cognitive operations over extended periods [3]. Statistical evidence indicates that human error, often driven by cognitive overload, remains a primary threat to aviation safety [4,5]. Consequently, keeping the cognitive workload (CW) of ATCOs within an optimal functional range is of paramount importance [6,7]. To mitigate the risks associated with operator overload, neuroadaptive support paradigms empowered by brain–computer interface (BCI) technologies have emerged as a promising solution. Such systems aim to continuously monitor operators’ cognitive states and dynamically adjust automation assistance policies in real time [8,9]. Within this context, the development of objective, accurate, and low-latency cognitive workload decoding (CWD) methods has become a critical prerequisite for facilitating closed-loop human-automation teaming in modern air traffic control (ATC) [10,11].

Among various physiological measurement modalities, electroencephalography (EEG) has been widely recognized as a robust biomarker for quantifying CW [12,13]. By capturing the synchronous electrical activity of neuronal populations directly from the scalp, EEG provides exceptional temporal resolution to reflect instantaneous workload fluctuations [14,15]. Despite the encouraging performance reported by prior machine learning and deep learning approaches in conventional closed-set CWD protocols [16,17,18], decoding the cognitive workload of ATCOs in realistic operational settings remains exceptionally challenging.

The primary obstacle stems from the multidimensional nature of ATC tasks and the resulting severe feature overlapping in scalp EEG signals. ATCO workload is not a unidimensional scalar; rather, it originates from diverse, intertwined cognitive demands [19]. Specifically, controllers must concurrently manage *information maintenance and integration* (e.g., memorizing callsigns and responding to voice communications) alongside *conflict monitoring and sequencing decisions* (e.g., visual–spatial trajectory prediction) [20,21,22]. These concurrent, heterogeneous cognitive processes activate distinct yet spatially overlapping cortical networks [23,24,25]. However, contemporary decoding models predominantly map distinct task demands into homogenous neural representations through conventional convolutional or recurrent architectures. Such methods treat electroencephalography signals as uniform Euclidean grids or sequences, thereby failing to capture the complex spatial correlations among distinct brain regions. Subsequent advancements introduced static graph neural networks to model these spatial dependencies [26,27,28]. Yet, static topologies remain inadequate for air traffic control scenarios where functional connectivity reorganizes rapidly in response to shifting cognitive demands.

More recently, dynamic graph neural networks have been adapted for neural signal processing to track these temporal variations. Existing dynamic frameworks predominantly construct time-evolving adjacency matrices relying entirely on data-driven mathematical optimizations. While mathematically flexible, these unconstrained statistical approaches lack domain-specific physiological priors. Consequently, they inherently generate opaque topological structures that struggle to disentangle concurrent and heterogeneous cognitive demands under high workload conditions. Because the human brain dynamically transitions among distinct functional sub-networks to allocate limited cognitive resources [29,30], relying on uninterpretable statistical correlations inevitably leads to severe feature confounding and degrades classification efficacy.

To address this fundamental limitation, it is essential to transcend the paradigm of treating neural signals as uniform sequences or purely mathematical dynamic graphs. A more scientifically rigorous approach involves embedding specific neurophysiological priors to explicitly decouple the mixed neural signals into independent topological sub-networks. By leveraging microstate theory as a structural constraint, the dynamic graph generation process is forced to align with genuine functional brain network reorganizations rather than opaque data-driven mappings. This paradigm shift from statistical connectivity to physiologically constrained topological disentanglement facilitates precise workload evaluation and establishes a mechanistic foundation for practical interventions.

Motivated by these challenges, this study proposes a Dynamic Multi-Graph Disentanglement Convolutional Network (DMG-GCN) for EEG-based cognitive workload decoding in air traffic control. The proposed architecture aims to adaptively disentangle complex, overlapping EEG signals into distinct microstate topological representations, thereby achieving both superior decoding performance and explicit neural interpretability. The main contributions of this study are summarized as follows:

(a) We propose the DMG-GCN architecture, a novel end-to-end deep learning framework that formulates the cognitive workload decoding task as a dynamic topological disentanglement problem, effectively resolving the severe feature confounding issue inherent in multidimensional air traffic control operations.
(b) We develop a dynamic multi-microstate topology generation mechanism integrated with a temporal–spectral feature extractor. This synergistic approach autonomously learns multiple distinct connectivity basis matrices from highly coupled EEG signals, ensuring the extraction of non-redundant functional sub-networks that adaptively capture dynamic neural transitions.
(c) Through comprehensive network visualization and statistical analyses, we suggest the neurophysiological interpretability of the microstate topologies learned by DMG-GCN. The model extracts dynamic neural reorganization patterns across varying workloads, encompassing the transition from global distributed monitoring to temporal multimodal integration and parietal–occipital parallel processing, thereby providing a theoretical foundation for future BCI developments.

The remainder of this paper is organized as follows: Section 2 introduces the dataset and experimental procedure. Section 3 presents the proposed DMG-GCN methodology. Section 4 reports the experimental setup and decoding results. Section 5 discusses the neurophysiological mechanisms and practical implications. Finally, Section 6 concludes the paper.

## 2. Experiment

### 2.1. Participants

An *a priori* power analysis was conducted using G*Power 3.1 software (α=0.05, expected power = 0.80). The calculation indicated that a minimum sample size of 15 participants is required to achieve statistical significance for within-subject comparisons [31]. This baseline size exceeds the typical staffing configuration of a single air traffic control shift at a medium-sized airport and provides sufficient sample support for feature disentanglement and workload modeling. To account for potential data loss and artifact contamination during EEG recordings, twenty-one healthy volunteers were recruited and successfully completed the entire EEG experiment. Specifically, this participant cohort consisted of specialized trainees who are currently enrolled as students majoring in air traffic management. These specialized trainees possessed the requisite theoretical background and operational familiarity to perform the simulated aerodrome control tasks, which were subsequently utilized to train and evaluate the proposed cognitive workload decoding model. All participants possessed normal or corrected-to-normal vision and hearing, reported no history of neurological or psychiatric disorders, and were free from long-term psychotropic medication use. Prior to the formal experiment, each participant received standardized task instructions and completed practice sessions until fully familiarized with the operational procedures, thereby minimizing extraneous cognitive load induced by interface unfamiliarity. Written informed consent was obtained from all participants after a comprehensive explanation of the study protocol.

### 2.2. Apparatus

**Figure 1:**
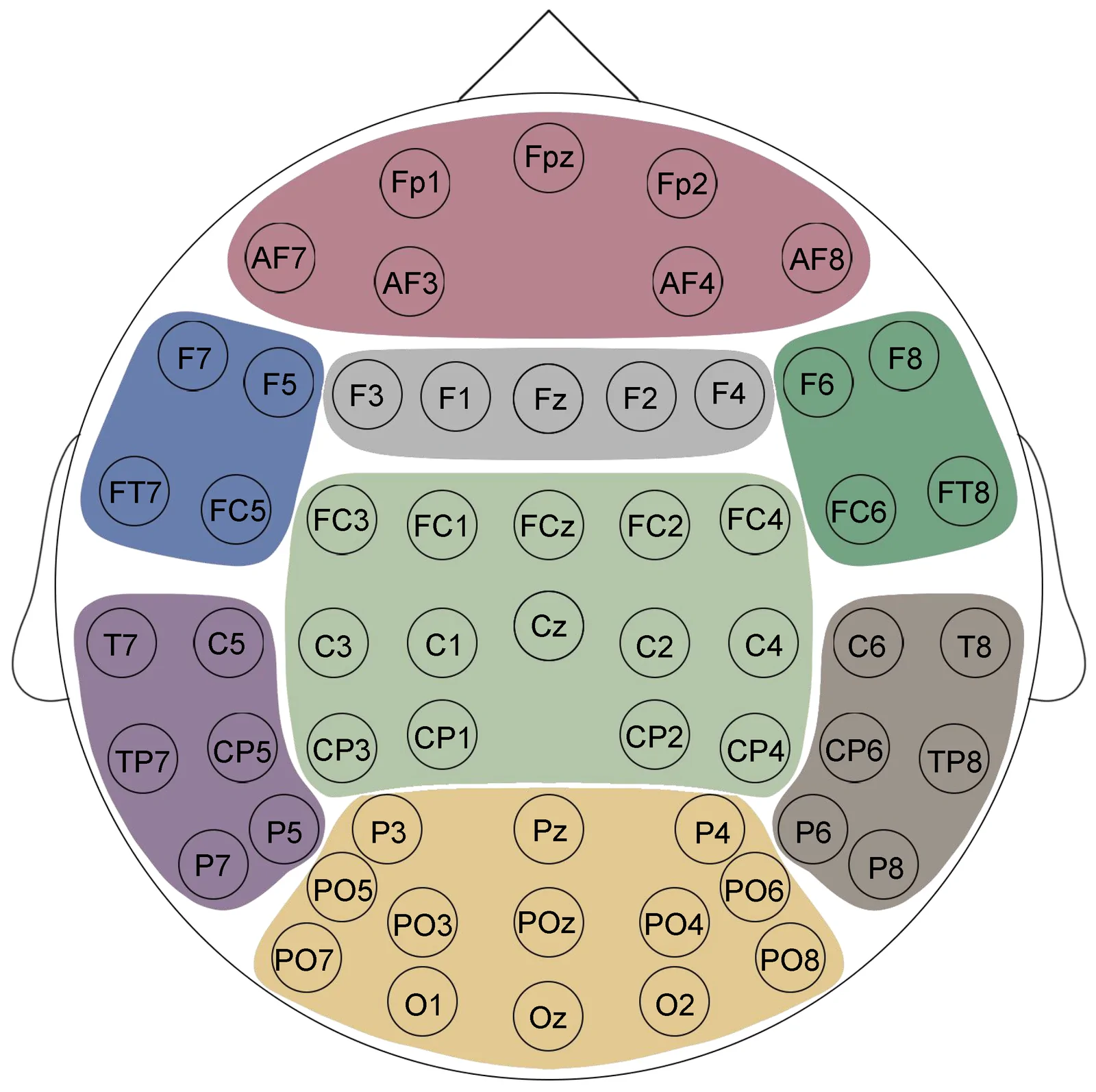
The distribution of electrode channels across different brain regions. Different colors represent different brain regions.

Continuous EEG data were recorded using a 64-channel wet-electrode system (NeuroHUB, Neuracle Technology, Shanghai, China). The electrodes were positioned according to the international 10-10 system, covering the frontal, parietal, occipital, and temporal regions. After excluding four electrooculogram (EOG) channels utilized for monitoring horizontal and vertical eye movements and one electrocardiogram (ECG) channel, 59 core EEG channels were retained for subsequent analysis. The spatial distribution of these 59 channels across the scalp is illustrated in Figure 1, and the specific electrodes corresponding to each brain region are detailed in Table 1. During signal acquisition, the data were synchronously transmitted to a workstation for real-time voltage monitoring. The reference electrode (REF) was placed at the occipital region, while the ground electrode (GND) was positioned at the midline frontal pole to ensure signal reference stability and robust interference suppression throughout the recording. All channel signals were online-referenced to this independent electrode and appropriately re-referenced during the preprocessing stage. The EEG signals were digitized at a sampling rate of 1000 Hz, and electrode impedances were strictly maintained below 10 kΩ.

### 2.3. Experimental Procedure

In conventional cognitive workload research, experimental paradigms are generally categorized into cognitive-oriented and operation-oriented approaches. The former (e.g., *n*-back, Sternberg working memory tasks) facilitates strict control over workload gradients but lacks ecological validity; the latter (e.g., simulated driving, Multi-Attribute Task Battery [MATB]) offers operational realism but struggles with precise, hierarchical workload regulation. Given the highly standardized and multitasking nature of air traffic control (ATC), generating realistic yet precisely controllable workload levels is uniquely challenging. To address this, we designed a simulated airport aerodrome control paradigm that integrates the advantages of both approaches. Compared to approach and area control, aerodrome control features precise temporal tags and highly standardized operational procedures, making it an ideal scenario for constructing a cross-subject contrastive learning database and evaluating the generalization robustness of deep learning models.

**Figure 2:**
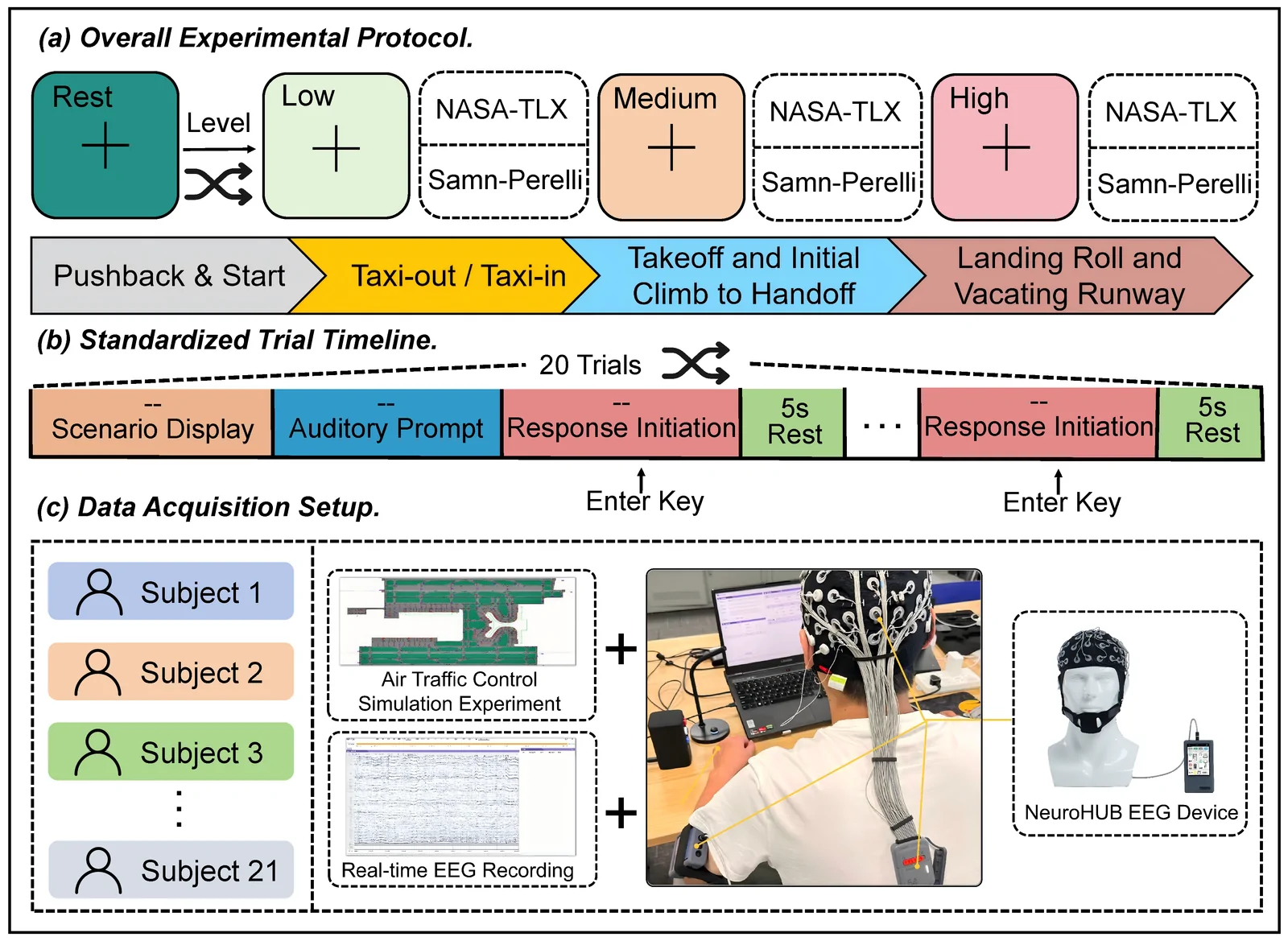
Experimental design and scenario illustrations. (**a**) Detailed setup for the cognitive task timeline. (**b**) Level design and examples of varying situational complexities. (**c**) An example trial executed at the Medium Workload (L2) difficulty level.

The experimental scenarios encompass five typical operational phases: pushback and startup, taxiing (inbound/outbound), runway holding/crossing, takeoff and initial climb, and landing roll and vacating. To focus on cross-subject workload prediction, emergency protocols were excluded. As illustrated in Figure 2, the cognitive workload was systematically modulated into three distinct tiers (Low, Medium, and High) by progressively escalating information density and situational complexity:

- Low Workload (L1): Key text information (e.g., aircraft callsigns, taxi routes, runway numbers) is displayed on the screen synchronously with voice prompts. Participants perform basic information matching and issue routine clearances, requiring minimal cognitive resources for situational integration and decision-making.
- Medium Workload (L2): Textual auxiliary information is completely removed, and physical recording tools are prohibited. Participants must rely entirely on auditory working memory to retain and process multi-aircraft state information internally. This significantly elevates the cognitive demands associated with information maintenance and internal integration.
- High Workload (L3): Building upon the voice-only condition, the scenarios introduce multiple aircraft with potential operational conflicts or converging taxiways. Participants must simultaneously monitor multi-target dynamics, predict potential conflicts, and sequence safe clearances (e.g., giving way, crossing) within the same trial. This pushes situational awareness and conflict-resolution demands to a peak level.

The standardized timeline for a single trial proceeds as follows: an aircraft operational situation is displayed for 10 seconds, followed by a scenario voice prompt. Under the High-Workload condition, a mental arithmetic task is immediately embedded post-prompt to measure reaction time and accuracy under peak load. After pressing the Enter key, the participant begins recording the voice clearance. The system logs the reaction time (from the end of the prompt to the start of recording) and the total recording duration. A 5-second rest period follows each trial to dissipate transient workload. Prior to the main experiment, a 1-minute eyes-closed resting-state baseline was recorded. Subsequently, the L1, L2, and L3 blocks were presented in a randomized order, with each block containing 20 independent, randomized trials to ensure the temporal precision of the workload labels. Brief rests were permitted between blocks to prevent cumulative fatigue. Post-experiment, participants completed the NASA-TLX subjective workload scale and the Samn–Perelli fatigue scale. The experiment was conducted in a standardized, quietly illuminated laboratory. To minimize electromyographic (EMG) artifacts, participants’ hands were fixed at a baseline position, and only minimal finger movements were allowed to press the Enter key.

### 2.4. Data Preprocessing

To guarantee the reliability of the extracted features, the raw EEG sequences were rigorously preprocessed using the open-source EEGLAB toolbox within the MATLAB R2024a environment. The preprocessing pipeline consisted of the following steps: (1) A 0.1 to 45 Hz FIR band pass filter was applied to eliminate low frequency DC drifts and high-frequency EMG interference. (2) A 50 Hz notch filter was utilized to remove power-line noise. (3) Independent Component Analysis (ICA) was performed to identify and reject typical artifact components, including eye blinks, ocular movements, and muscle activities, based on their spatial topographies and temporal waveforms. (4) The continuous EEG data for each participant were precisely aligned with the task phases and rest periods using electronic log timestamps, and subsequently segmented into independent single-trial blocks according to the workload conditions.

### 2.5. Implementation Details

The model development and performance evaluation were conducted on a 64-bit Windows 11 operating system equipped with a 14th Gen Intel Core i7-14650HX (5.20 GHz) CPU and an NVIDIA GeForce RTX 4060 GPU. The algorithms were developed in a Python 3.11 environment, utilizing the PyTorch (version 2.0.1) deep learning framework for model training and inference. To rigorously assess the cognitive workload prediction capability across subjects while strictly preventing any subject level data leakage, a leave-one-out cross-validation strategy was implemented. Specifically, in the *i*-th fold, all samples from the *i*-th subject strictly served as the isolated test set. Two subjects were randomly selected from the remaining twenty subjects to form the validation set, and the data from the other eighteen subjects constituted the training set. To absolutely guarantee zero data leakage, all preprocessing and standardization procedures were executed independently for each subject prior to cross-validation, ensuring that no statistical distribution information from the test subject inadvertently influenced the training or validation phases. The network was trained for a maximum of two hundred epochs, incorporating an early stopping mechanism where training was terminated if the validation loss failed to decrease for twenty consecutive epochs to prevent overfitting.

To ensure a fair and reproducible evaluation, all deep learning baseline models alongside the proposed framework were implemented in Python utilizing the PyTorch framework and trained under a unified experimental protocol. The models were optimized using the Adam optimizer, with the initial learning rate determined via a grid search within the range of [0.0001, 0.005] on the validation set. A standard batch size of 64 was applied across all experimental runs. To prevent overfitting and ensure objective comparisons, all neural network architectures incorporated an identical early stopping mechanism, terminating the training process if the validation loss failed to decrease for 20 consecutive epochs.

Furthermore, the hyperparameters for each baseline model were systematically optimized to reflect their representative capabilities on our specific dataset. For the traditional Support Vector Machine, a Radial Basis Function kernel was employed, with the penalty parameter *C* and kernel coefficient γ optimized via grid search (C∈{0.1,1,10}, γ∈{0.01,0.1,1}). For EEGNet, the temporal and spatial filter parameters were configured to the standard optimal settings of F1=8, D=2, and F2=16. The EEG-Conformer utilized 4 attention heads and a transformer depth of 2. For ASTGCN, the Chebyshev polynomial order was set to 3, and the model contained 2 spatiotemporal blocks. The LMDA-Net channel depth was configured to 9 with an attention reduction ratio of 4. Documenting this systematic optimization verifies that the reported performance gains of the proposed framework stem from its structural methodological advantages rather than disproportionate hyperparameter tuning.

## 3. Method

**Figure 3:**
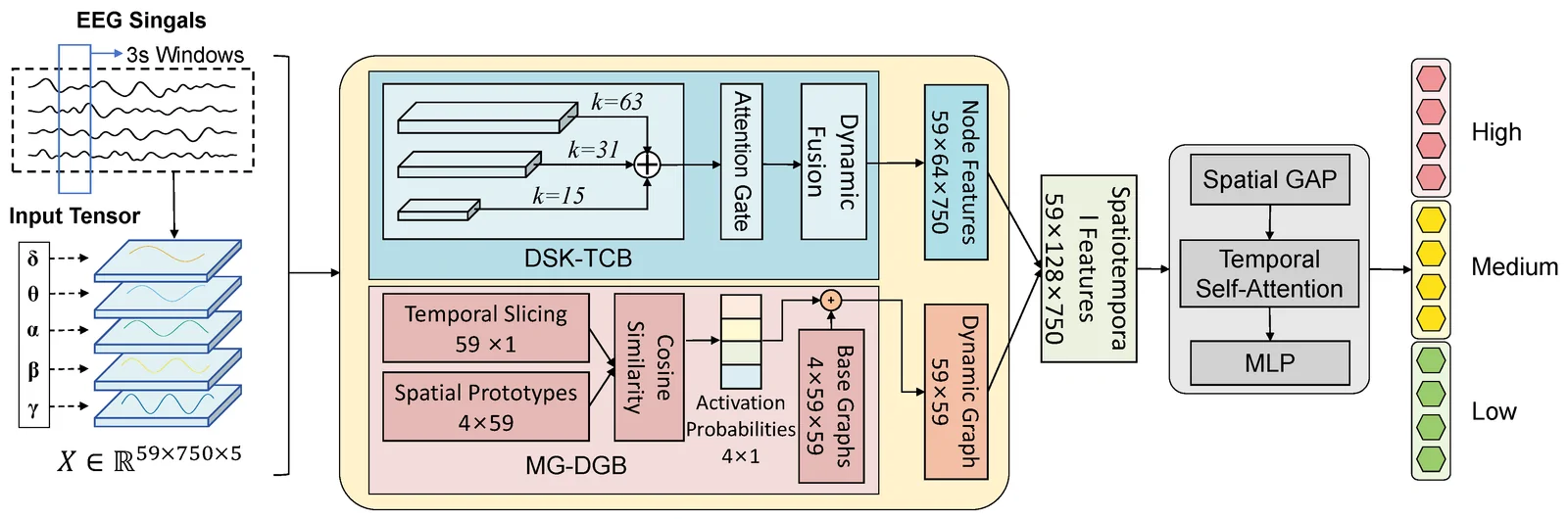
The overall architecture of the proposed Dynamic Microstate-Guided Graph Convolutional Network (DMG-GCN).

To address complex inter-subject variability in air traffic controller (ATCO) cognitive workload (CW) decoding, this paper proposes a Dynamic Microstate-Guided Graph Convolutional Network (DMG-GCN). The DMG-GCN framework aims to learn representations that are robust to subject shifts and highly sensitive to high-workload features by deeply integrating multi-scale frequency domain features with micro-topological connectivity. Specifically, the end-to-end information flow of the network consists of four core stages, as illustrated in Figure 3.

First, a sample generation module segments continuous multi-subject EEG signals into fixed-length time windows and extracts multi-band sequences to construct structured spatiotemporal-frequency input tensors. Second, the network enters a parallel dual-branch processing stage: the Feature Stream utilizes a Dynamic Selective Kernel Temporal Convolutional Block (DSK-TCB) to adaptively extract multi-scale local temporal–spectral dynamics from each electrode node, while the Topology Stream utilizes a Microstate-Guided Dynamic Graph Block (MG-DGB) to generate a dynamic graph adjacency matrix representing global instantaneous functional connectivity based on learnable microstate prototype graphs and real-time activation probabilities. Subsequently, a Spatiotemporal Dynamic Graph Convolution module uses the microstate topology as a routing matrix to perform spatial-level graph aggregation on the multi-scale node features, achieving deep synergy between neural topological structures and spectral characteristics. Finally, a Global Feature Integration and Classification Decision module focuses on high-workload transition moments via a temporal self-attention mechanism and performs CW level classification combined with Label Smoothing regularization, effectively mitigating gradient noise caused by subjective labeling.

### 3.1. Data Representation and Sample Generation

The primary objective of this stage is to construct input tensors that explicitly reflect multi-band spatiotemporal characteristics and to define a standardized physical node space for the subsequent graph convolutional network.

Let the continuous EEG signals from a single trial be denoted as a matrix $E\in R^{C\times T_{trial}}$, where C=59 represents the number of effective scalp electrode channels retained after the removal of irrelevant channels (e.g., EOG and ECG), and $T_{trial}$ denotes the total number of sampling points in the trial. During preprocessing, the signals are downsampled to a sampling rate of $f_{s}=250\;\mathrm{Hz}$. To capture the quasi-stationary characteristics of cognitive states while satisfying the low-latency requirements for continuous monitoring, the continuous signals are segmented into overlapping time windows. Based on the neurophysiological properties of EEG slow-wave rhythms, the window length is set to K=3s (i.e., 750 sampling points) with a sliding step of Δ=1s. This specific duration was selected to balance frequency resolution and cognitive state stationarity. A 3-second window provides adequate temporal extension to reliably capture low-frequency oscillations, while remaining sufficiently concise to preserve the quasi-stationarity assumption of transient cognitive dynamics during operational tasks.

For the *i*-th segmented raw window sample $x_{i}\in R^{C\times K}$, a Butterworth band-pass filter bank is applied to decompose the broadband signal into F=5 classical neural frequency bands: δ (1–3 Hz), θ (4–7 Hz), α (8–13 Hz), β (13–30 Hz), and γ (30–45 Hz). Following frequency decomposition, the components are stacked along a new dimension to reconstruct the sample into a high-dimensional tensor $X_{i}\in R^{C\times K\times F}$. This tensor embeds rich physiological information across 59 spatial nodes, 750 time steps, and 5 spectral bands, serving as the direct input for the downstream dual-branch network.

In the context of supervised learning, each window sample $X_{i}$ is assigned a discrete cognitive workload label $y_{i}\in \left\{0,1,2\right\}$, mapping strictly to the low, medium, and high ATC workload levels objectively defined by the experimental paradigm. Additionally, to track the subject origin during the training phase, each sample is associated with a subject identifier $v_{i}\in \left\{1,\ldots ,21\right\}$. During model optimization, *B* window samples are randomly sampled to form a mini-batch tensor $X\in R^{B\times C\times K\times F}$. This unified data representation provides a precise mathematical foundation for node feature extraction and dynamic whole-brain network computation.

### 3.2. Parallel Dual-Branch Feature Extraction Architecture

Upon receiving the initial spatiotemporal-frequency tensor X, the network routes the data into a parallel dual-branch architecture. To simplify mathematical notations, the batch size dimension *B* is omitted in the subsequent derivations, and operations are described for a single sample $X_{i}\in R^{C\times K\times F}$.

#### 3.2.1. Dynamic Selective Kernel Temporal Convolutional Block (DSK-TCB)

**Figure 4:**
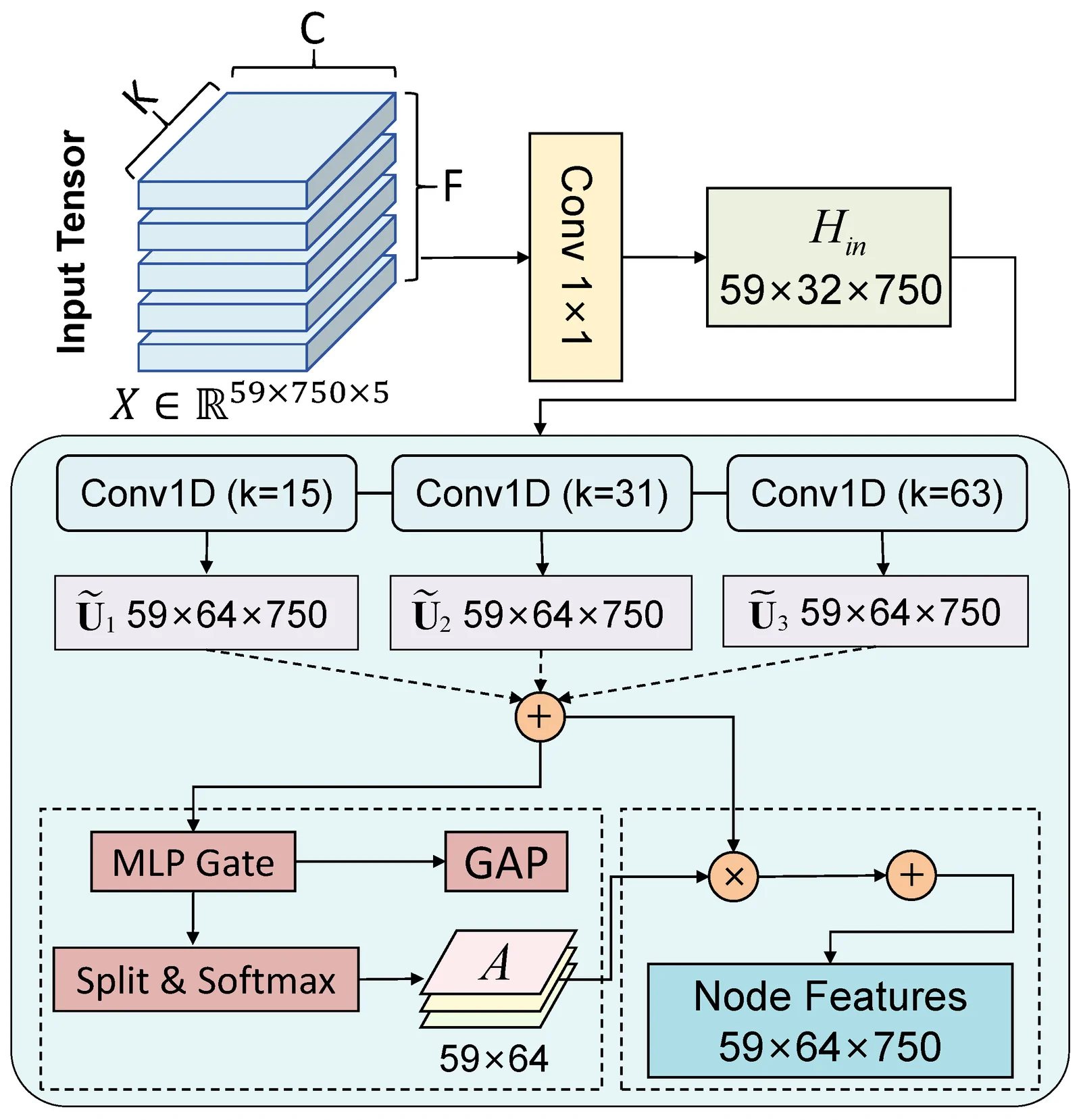
Detailed architecture of the Dynamic Selective Kernel Temporal Convolutional Block (DSK-TCB).

ATCOs exhibit highly non-stationary EEG characteristics when processing tasks of varying cognitive difficulties. To adaptively adjust the temporal receptive field based on the spectral characteristics of the input signal, we propose the Dynamic Selective Kernel Temporal Convolutional Block (DSK-TCB), as detailed in Figure 4.

First, the input tensor $X_{i}$ is projected along the frequency dimension *F* using a 1×1 convolution layer to fuse the multi-band information, yielding a node-level input feature matrix $H_{in}\in R^{C\times D_{in}\times K}$, where $D_{in}$ is the initial feature channel dimension. Subsequently, the network passes $H_{in}$ through three parallel 1D temporal convolutional branches with varying kernel sizes $k_{1}=15,\;k_{2}=31$, and $k_{3}=63$. Given the 250 Hz sampling rate, these kernel sizes establish physical temporal receptive fields of 60, 124, and 252 ms. This multi-scale configuration is systematically designed to capture the diverse spectral dynamics of neural signals, where smaller kernels focus on transient high-frequency variations and larger kernels extract sustained low-frequency physiological trends. Zero-padding is applied to ensure the temporal length *K* remains unchanged. The output feature tensors of these branches are denoted as ${\tilde{U}}_{1},{\tilde{U}}_{2},{\tilde{U}}_{3}\in R^{C\times D\times K}$, where *D* is the unified feature channel dimension.

To guide the network in perceiving the global temporal–spectral distribution of local nodes, the feature tensors from the three branches are element-wise summed to obtain an aggregated feature tensor $U={\tilde{U}}_{1}+{\tilde{U}}_{2}+{\tilde{U}}_{3}$. Global Average Pooling (GAP) is then applied along the time dimension *K* to extract a statistical matrix $s\in R^{C\times D}$ representing the overall energy of each node. Specifically, for the *d*-th feature channel of the *c*-th electrode node, the global statistic $s_{c,d}$ is calculatedas

(1) $$s_{c,d}=\frac{1}{K}\sum_{t=1}^{K}U_{c,d,t}$$

A two-layer Multi-Layer Perceptron (MLP) acts as an attention gating mechanism and is applied independently to the statistical vector $s_{c}\in R^{D}$ of each node *c* to compute receptive field allocation weights:

(2) $$z_{c}=W_{2}\delta \left(B\left(W_{1}s_{c}\right)\right)$$

where $W_{1}\in R^{\frac{D}{r}\times D}$ and $W_{2}\in R^{3D\times \frac{D}{r}}$ are learnable weight matrices, *r* is the channel reduction ratio to limit model complexity, B(·) denotes Batch Normalization, and δ(·) is the ReLU activation function.

The output vector $z_{c}\in R^{3D}$ is uniformly split into three sub-vectors $\left[z_{c,1},z_{c,2},z_{c,3}\right]$, each belonging to $R^{D}$. A channel-wise Softmax function is applied across the branches to generate normalized dynamic weight matrices $A_{1},A_{2},A_{3}\in R^{C\times D}$. For instance, the attention weight for the *d*-th channel of the 1-st branch is computed as $A_{1}\left[c,d\right]=\frac{\exp(z_{c,1,d})}{\sum_{j=1}^{3}\exp\left(z_{c,j,d}\right)}$. The final temporally fused node feature tensor $H_{node}\in R^{C\times D\times K}$ is obtained by computing the weighted sum of the multi-scale features:

(3) $$H_{node}=\left(A_{1}⊙{\tilde{U}}_{1}\right)+\left(A_{2}⊙{\tilde{U}}_{2}\right)+\left(A_{3}⊙{\tilde{U}}_{3}\right)$$

where ⊙ denotes the Hadamard product, and the attention matrices $A_{j}$ are explicitly broadcasted along the temporal dimension *K* during multiplication.

#### 3.2.2. Microstate-Guided Dynamic Graph Block (MG-DGB)

**Figure 5:**
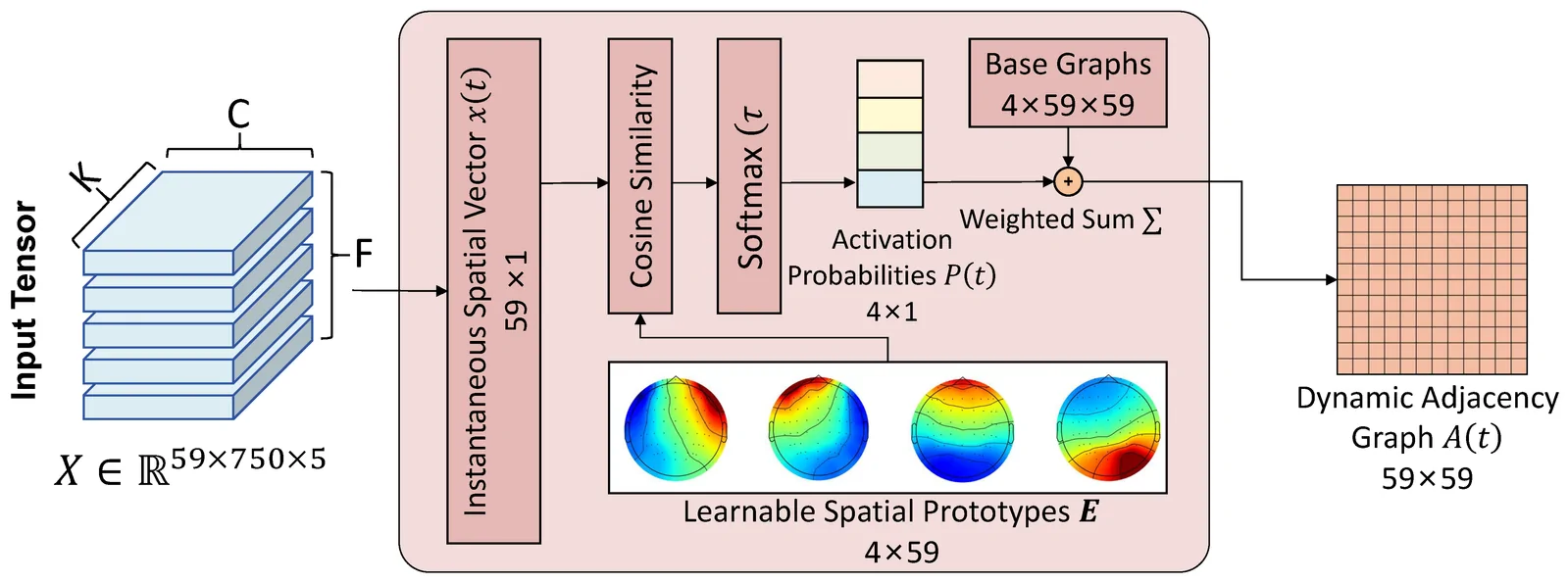
Detailed illustration of the Microstate-Guided Dynamic Graph Block (MG-DGB).

To address topological distribution shifts across subjects, this framework introduces EEG microstate theory as a physiological prior to dynamically construct a time-evolving graph adjacency matrix $A\left(t\right)\in R^{C\times C}$, as depicted in Figure 5.

The network defines and maintains $N_{m}=4$ learnable spatial prototype vectors inspired by microstate theory $E_{m}\in R^{C\times 1}$ alongside their corresponding base functional connectivity matrices $A_{m}\in R^{C\times C}$ ($m\in \{1,\ldots ,N_{m}\}$). The number of prototypes is set to 4 to conceptually align with classical electroencephalography microstate theory. Extensive neurophysiological research indicates that spontaneous scalp potential topographies predominantly cluster into four fundamental canonical classes. By utilizing this established physiological prior, we ensure that the network possesses sufficient representational capacity to disentangle functional subnetworks while mitigating the risk of introducing excessive computational redundancy. However, it is essential to clarify that these learnable spatial prototypes represent data-driven functional approximations optimized specifically for cognitive workload decoding. Consequently, they should not be strictly equated with canonical resting state microstates extracted via conventional statistical clustering algorithms. Unlike traditional offline clustering methodologies, the proposed framework identifies these functional microstates through an end to end learnable mechanism. The network dynamically calculates the cosine similarity between the instantaneous broadband spatial topography and the learned prototype vectors. By converting these similarities into real-time activation probabilities via a temperature-scaled transformation, the model continuously identifies the dominant microstate configuration at any specific temporal step in a purely data-driven manner. At any given time step t∈{1,…,K}, the instantaneous broadband spatial topography vector $x\left(t\right)\in R^{C\times 1}$ is extracted from the raw window sample $x_{i}$. The network calculates the cosine similarity between x(t) and each prototype $E_{m}$, mapping these similarities into real-time activation probabilities $p_{m}\left(t\right)$ using a Softmax function scaled by a temperature coefficient τ:

(4) $$p_{m}\left(t\right)=\frac{\exp(\mathrm{sim}\left(x\left(t\right),E_{m}\right)/\tau )}{\sum_{j=1}^{N_{m}}\exp\left(\mathrm{sim}\left(x\left(t\right),E_{j}\right)/\tau \right)}$$

where $\mathrm{sim}\left(a,b\right)=\frac{a^{T}b}{∥a∥∥b∥}$. The dynamic functional connectivity graph $A\left(t\right)\in R^{C\times C}$ at time step *t* is formulated as the probability-weighted expectation of all base connectivity matrices:

(5) $$A\left(t\right)=\sum_{m=1}^{N_{m}}p_{m}\left(t\right)A_{m}$$

### 3.3. Spatiotemporal Dynamic Graph Convolutional Fusion Layer

This layer systematically integrates the local multi-scale spectral features $H_{node}$ with the global dynamic topological structures A(t). For a specific time step *t*, we define the instantaneous adjacency matrix with self-connections as $\tilde{A}\left(t\right)=A\left(t\right)+I_{C}$ (where $I_{C}\in R^{C\times C}$ is the identity matrix), and its corresponding diagonal degree matrix as $\tilde{D}\left(t\right)$ with ${\tilde{D}}_{c,c}\left(t\right)=\sum_{j}{\tilde{A}}_{c,j}\left(t\right)$.

For the *l*-th graph convolutional layer (l∈{0,…,L−1}), the spatial feature propagation is executed as follows:

(6) $$Z^{\left(l+1\right)}\left(t\right)=\sigma (\tilde{D}{\left(t\right)}^{-\frac{1}{2}}\tilde{A}\left(t\right)\tilde{D}{\left(t\right)}^{-\frac{1}{2}}Z^{\left(l\right)}\left(t\right)W^{\left(l\right)})$$

where the initial node feature is set as $Z^{\left(0\right)}\left(t\right)=H_{node}\left(:,:,t\right)\in R^{C\times D}$. $W^{\left(l\right)}\in R^{D^{\left(l\right)}\times D^{\left(l+1\right)}}$ is a layer-specific learnable weight matrix, and σ(·) represents the ReLU activation function. After *L* layers of message passing, the temporal slices are stacked to form the final spatiotemporal graph convolutional feature tensor $Z_{gcn}\in R^{C\times D_{out}\times K}$, where $D_{out}=D^{\left(L\right)}$.

### 3.4. Global Feature Integration and Classification Decision

The final module condenses the high-dimensional node-level tensor $Z_{gcn}$ into a singular global representation to execute the CW level classification.

Initially, the network applies spatial Global Average Pooling across the node dimension *C* to yield a temporal sequence matrix $H_{seq}\in R^{D_{out}\times K}$. Let $h_{t}\in R^{D_{out}}$ denote the feature vector at time *t*. Recognizing that high-workload markers may only manifest during brief critical transitions (e.g., sudden conflict alerts), a temporal self-attention mechanism is employed to calculate normalized importance weights $\alpha_{t}$ for each frame:

(7) $$e_{t}=v^{T}\tanh\left(W_{att}h_{t}+b_{att}\right),\;\alpha_{t}=\frac{\exp(e_{t})}{\sum_{k=1}^{K}\exp\left(e_{k}\right)}$$

where $W_{att}\in R^{d_{att}\times D_{out}}$ and $b_{att}\in R^{d_{att}}$ are the transformation matrix and bias of the attention layer, respectively, and $v\in R^{d_{att}}$ is the context vector used to score the hidden representation. The global cognitive feature vector $h_{final}\in R^{D_{out}}$ is then computed via a weighted summation: $h_{final}=\sum_{t=1}^{K}\alpha_{t}h_{t}$.

The prediction probability distribution $\hat{y}\in R^{N_{c}}$ over the $N_{c}=3$ workload classes is derived through a linear classification head:

(8) $$\hat{y}=\mathrm{Softmax}\left(W_{c}h_{final}+b_{c}\right)$$

where $W_{c}\in R^{N_{c}\times D_{out}}$ and $b_{c}\in R^{N_{c}}$ map the final features to the logit space.

To prevent the model from becoming overconfident and to mitigate gradient noise caused by inherent subjectivity in ATCO workload labeling, Label Smoothing regularization is introduced. The true one-hot encoded label $y_{hard}\in {\left\{0,1\right\}}^{N_{c}}$ is transformed into a soft label distribution $y_{smooth}\in R^{N_{c}}$:

(9) $$y_{smooth}=\left(1-\epsilon \right)y_{hard}+\frac{\epsilon }{N_{c}}1$$

where ϵ∈(0,1) is the smoothing hyperparameter and $1\in R^{N_{c}}$ is a vector of ones. The entire DMG-GCN framework is optimized end-to-end using the smoothed cross-entropy loss function:

(10) $$L=-\sum_{i=1}^{N_{c}}y_{smooth,i}\log\left({\hat{y}}_{i}\right)$$

In summary, the DMG-GCN architecture achieves a rigorously decoupled extraction of local multi-scale temporal–spectral dynamics and global microstate topological structures. By leveraging temporal attention to highlight task-critical transitions and suppressing subjective label noise via Label Smoothing, the proposed framework establishes a mathematically sound and robust mapping from raw multi-band EEG signals to discrete cognitive workload states.

## 4. Results

### 4.1. Behavioral Validation of the Experimental Paradigm

To verify that the simulated air traffic control paradigm successfully induced distinct and escalating cognitive demands, we statistically analyzed the subjective ratings collected from the 21 subjects during the formal experiments. The cognitive workload was evaluated using the NASA-TLX scale (ranging from 0 to 100), while the subjective fatigue level was assessed via the Samn–Perelli scale (ranging from 1 to 7).

The statistical results exhibit a consistent hierarchical escalation across the three designed scenarios. Under the Low-Workload condition, the average NASA-TLX score was 47.60 (±9.56), and the Samn–Perelli fatigue score was 1.84 (±0.69). During the Medium-Workload task, these scores increased to 58.98 (±10.04) and 2.58 (±0.96), respectively. In the High-Workload scenario, the metrics peaked at a NASA-TLX score of 70.77 (±12.46) and a fatigue score of 3.63 (±1.07). Repeated measures statistical analyses reveal that the incremental increases between any two workload levels evaluated on both the NASA-TLX and the Samn–Perelli scales are highly significant (p<0.001). These behavioral assessments support the efficacy of the experimental design, suggesting that the varying situational complexities successfully modulated the subjects’ cognitive states, thereby providing a reliable behavioral ground truth for the subsequent neurophysiological decoding tasks.

### 4.2. Performance Comparison with State-of-the-Art Models

To comprehensively evaluate the performance of the proposed DMG-GCN model in air traffic control (ATC) cognitive workload decoding tasks, this study conducts comparative experiments with five representative baseline models. These models span a diverse methodological spectrum ranging from traditional machine learning to advanced deep graph learning, specifically including (1) SVM [32], a conventional machine learning classifier based on power spectral density (PSD) features; (2) EEGNet [33], a widely adopted lightweight spatiotemporal convolutional neural network for EEG processing; (3) EEG-Conformer [34], an architectural framework combining local feature extraction via convolutional neural networks with global attention mechanisms via Transformers; (4) ASTGCN [35], an attention-based spatiotemporal graph convolutional network; and (5) LMDA-Net [36], a recently proposed lightweight multi-dimensional attention network for EEG decoding. All models are evaluated using a unified training and testing protocol on the same dataset of 21 subjects. The quantitative performance metrics of each model are summarized in Table 2.

As indicated by the statistical results in Table 2, the proposed DMG-GCN consistently achieves the optimal performance across all three workload levels and evaluation metrics. Its overall average accuracy reaches 80.30%, representing an absolute improvement of 3.61% compared to the second-best baseline model, LMDA-Net (76.69%).

To rigorously evaluate whether the observed performance gains represent genuine methodological advancements rather than random variance, we conducted non-parametric Wilcoxon signed-rank tests on the subject-level overall average classification accuracies. This non-parametric test was selected because accuracy distributions across cross-validation folds frequently violate the normality assumption required for standard parametric testing. The statistical analysis reveals that, in terms of overall average accuracy, the performance improvement of the proposed DMG-GCN over the second-best baseline model, LMDA-Net, is statistically significant (p=0.004). Furthermore, the performance advantages of DMG-GCN over ASTGCN, EEG-Conformer, EEGNet, and SVM are all highly significant (p<0.001). Extended statistical evaluation on class-specific metrics further indicates that the proposed framework maintains a statistically meaningful advantage even in the highly confounded medium workload scenario. These statistical results suggest that the integration of the multi-microstate topological disentanglement mechanism provides a reliable enhancement in decoding efficacy.

An analysis of the performance under distinct workload conditions reveals a general decline in classification metrics across all models during the medium-workload task. For instance, the F1-score of LMDA-Net drops to 70.06% under the medium-workload condition. From a neuroscientific perspective, the medium-workload state corresponds to a transitional phase in the dynamic allocation of cortical cognitive resources, leading to feature overlapping in its EEG representations with both low- and high-workload states. Benefiting from the innovative multi-microstate topological disentanglement mechanism, DMG-GCN maintains a high F1-score of 73.18% under this condition, thereby effectively mitigating the adverse impact of feature confounding on classification performance.

**Figure 6:**
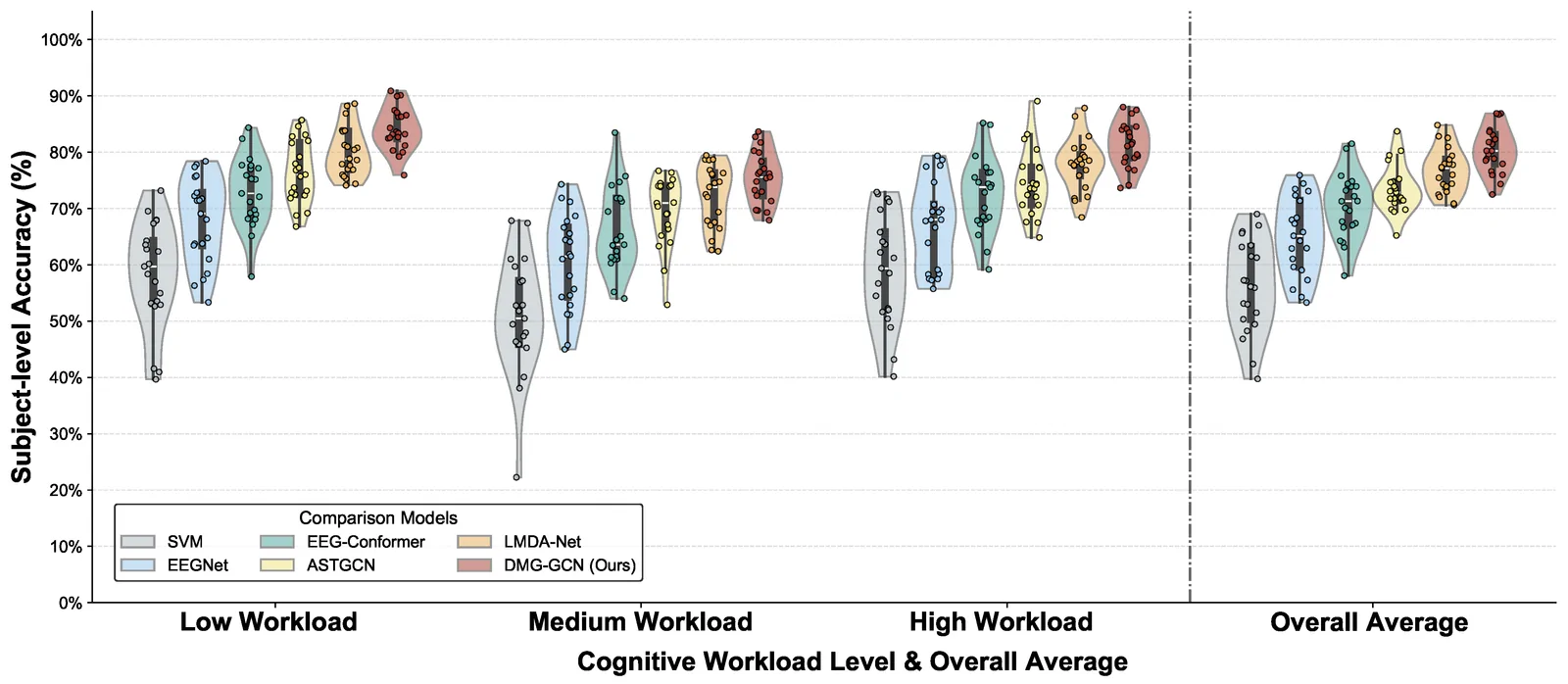
Kernel density estimation distributions and individual subject-level accuracy scatter plots of cross-subject classification accuracy across different cognitive workloads for all evaluated models.

To further investigate the stability of the models in cross-subject scenarios, Figure 6 presents the kernel density estimation (KDE) distributions overlaid with individual subject-level accuracy scatter plots under different workload conditions. Within this visualization, the vertical position of the distribution profile indicates the decoding accuracy level, whereas the width and dispersion of the distribution shape map the magnitude of cross-subject variance.

As illustrated in Figure 6, the data distributions of SVM and EEGNet exhibit pronounced long-tail characteristics, with overall accuracy standard deviations of 8.79% and 7.15%, respectively. This observation indicates that conventional handcrafted feature extraction and standard convolutional operations are highly sensitive to the individual neurophysiological variations among the participants, thereby limiting the generalization capability of these models across certain subjects. Although EEG-Conformer (incorporating self-attention mechanisms) and ASTGCN (utilizing spatiotemporal graph convolutions) improve the overall accuracy, they still manifest noticeable cross-subject performance fluctuations. In contrast, the data points of DMG-GCN (represented by the dark red regions) are significantly more concentrated across all categories, with its overall accuracy standard deviation constrained to 4.05%. This specific data distribution pattern demonstrates that DMG-GCN stably extracts the intrinsic microstate reorganization principles associated with cognitive workload transitions. By simultaneously maintaining high decoding accuracy and low cross-subject variance, DMG-GCN demonstrates superior robustness for cross-subject deployment.

### 4.3. Ablation Study and Architectural Analysis

This section aims to objectively evaluate the specific contributions of the core architectural mechanisms within the DMG-GCN model, specifically quantifying the individual efficacy of the dynamic selective kernel temporal convolutional block (DSK-TCB), the microstate-guided dynamic graph block (MG-DGB), and the temporal self-attention module toward the final cognitive workload decoding performance. To ensure the strict rigor of the controlled variable method, a comprehensive set of structural model variants was designed for rigorous cross-validation:

1. Base GCN: The microstate topology generator is completely removed, degrading the framework into a conventional spatiotemporal graph network that utilizes a global static learnable adjacency matrix.
2. w/o Microstate: The dynamic graph generation mechanism is retained, but the number of subgraphs is strictly restricted to $N_{m}=1$, thereby stripping away the multi-microstate topological representation capability.
3. w/o Temporal Conv: The multi-scale temporal extraction is replaced with a standard single-scale 1D convolution, evaluating the necessity of capturing diverse spectral dynamics.
4. w/o Attention: The temporal self-attention mechanism is removed in favor of standard global average pooling, assessing its specific role in focusing on task-critical transition moments.
5. w/o Label Smoothing: The network is optimized using standard cross-entropy without label softening, verifying its efficacy in mitigating subjective label noise.
6. DMG-GCN (Ours): The complete model proposed in this study.

**Figure 7:**
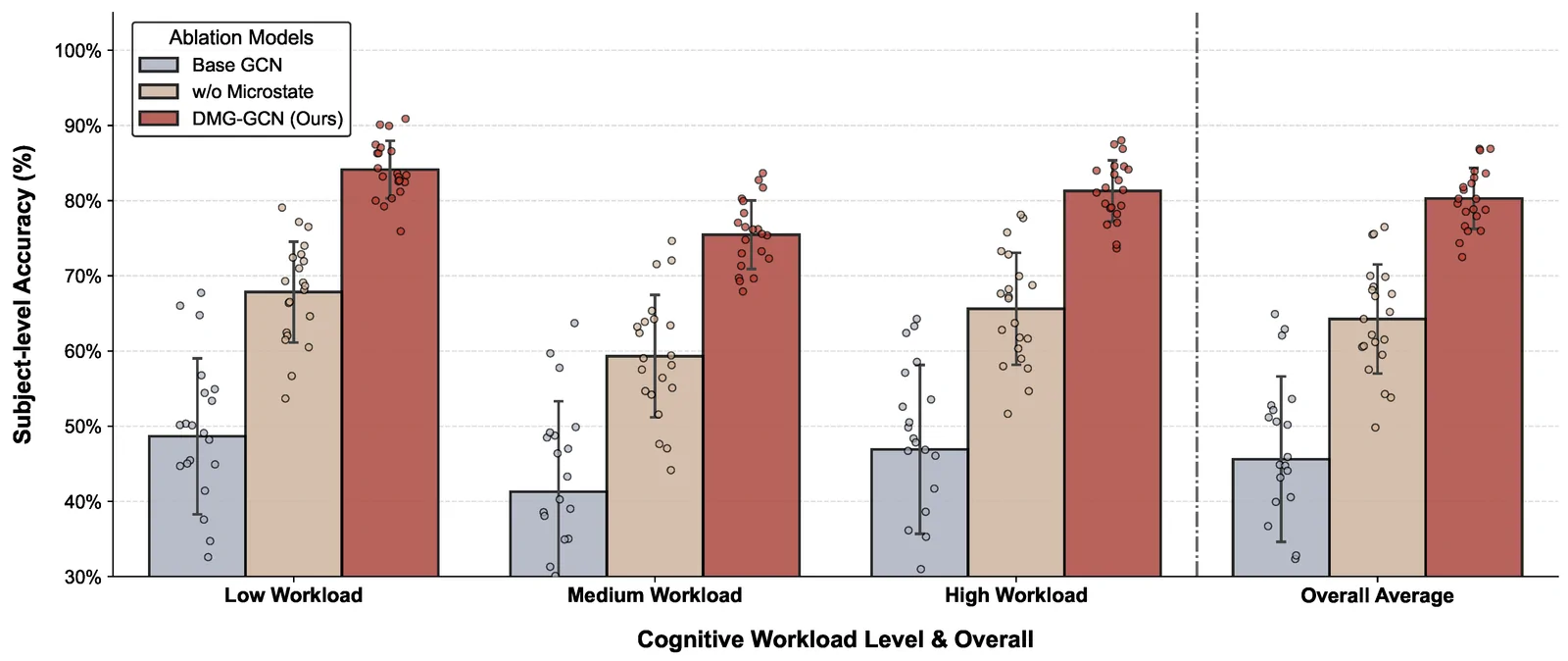
Subject-level accuracy distribution of ablation model variants across different cognitive workloads. The bar charts indicate the mean accuracy, with error bars representing the standard deviation and individual scatter points illustrating the true distribution of cross-subject performance.

The quantitative evaluation results of the 21 subjects across low-, medium-, and high-cognitive workloads for all structural variants are comprehensively summarized in Table 3. Additionally, the cross-subject accuracy distributions for the core topological variants are visually presented in Figure 7.

As illustrated in Figure 7 and Table 3, the complete DMG-GCN model achieves optimal decoding performance across all cognitive workload levels. In contrast, Base GCN exhibits severe representational collapse, with its overall accuracy decreasing substantially to 45.62% alongside significant cross-subject variance. This performance disparity demonstrates that conventional static graph structures fail to adaptively capture the rapidly shifting functional brain network connectivity inherent in complex operational air traffic control tasks.

Furthermore, the substantial performance gap between w/o Microstate and DMG-GCN validates the necessity of the proposed multi-representation disentanglement mechanism. Constrained by a single dynamic graph structure, the w/o Microstate model exhibits limitations in distinguishing the medium workload (F1-score decreasing to 56.87%), which represents a highly dynamic transitional phase of cognitive resources. Conversely, by relying on orthogonal microstate topologies, DMG-GCN effectively disentangles highly coupled brain signals, maintaining a medium workload F1-score of 73.18%. This indicates that fluctuations in specific cognitive demands drive the topology reorganization of distinct functional sub-networks.

The expanded structural ablation detailed in Table 3 further supports the efficacy of the remaining modules. Replacing the DSK-TCB with a standard convolution (w/o Temporal Conv) causes the overall accuracy to decrease to 72.45%, explicitly demonstrating the necessity of capturing diverse spectral dynamics. Removing the temporal attention mechanism (w/o Attention) decreases the overall accuracy to 76.18%, indicating the importance of focusing on task-critical transition moments. Additionally, omitting the label smoothing regularization results in a performance degradation to 78.54%, which validates its role in mitigating gradient noise caused by subjective labeling.

In addition to evaluating the architectural components, a systematic parameter sensitivity analysis was conducted to assess the influence of the hyperparameter defining the number of microstate prototypes ($N_{m}$). Empirical evaluations utilizing 2, 6, and 8 prototypes yielded overall accuracies of 73.12%, 79.15%, and 77.42%, respectively. These empirical results demonstrate that configuring exactly four prototypes ($N_{m}=4$) provides the optimal parameter balance. This specific configuration successfully captures the fundamental cognitive sub-networks without introducing severe model overfitting or unnecessary computational redundancy.

### 4.4. Visualization Analysis of Feature Representations

**Figure 8:**
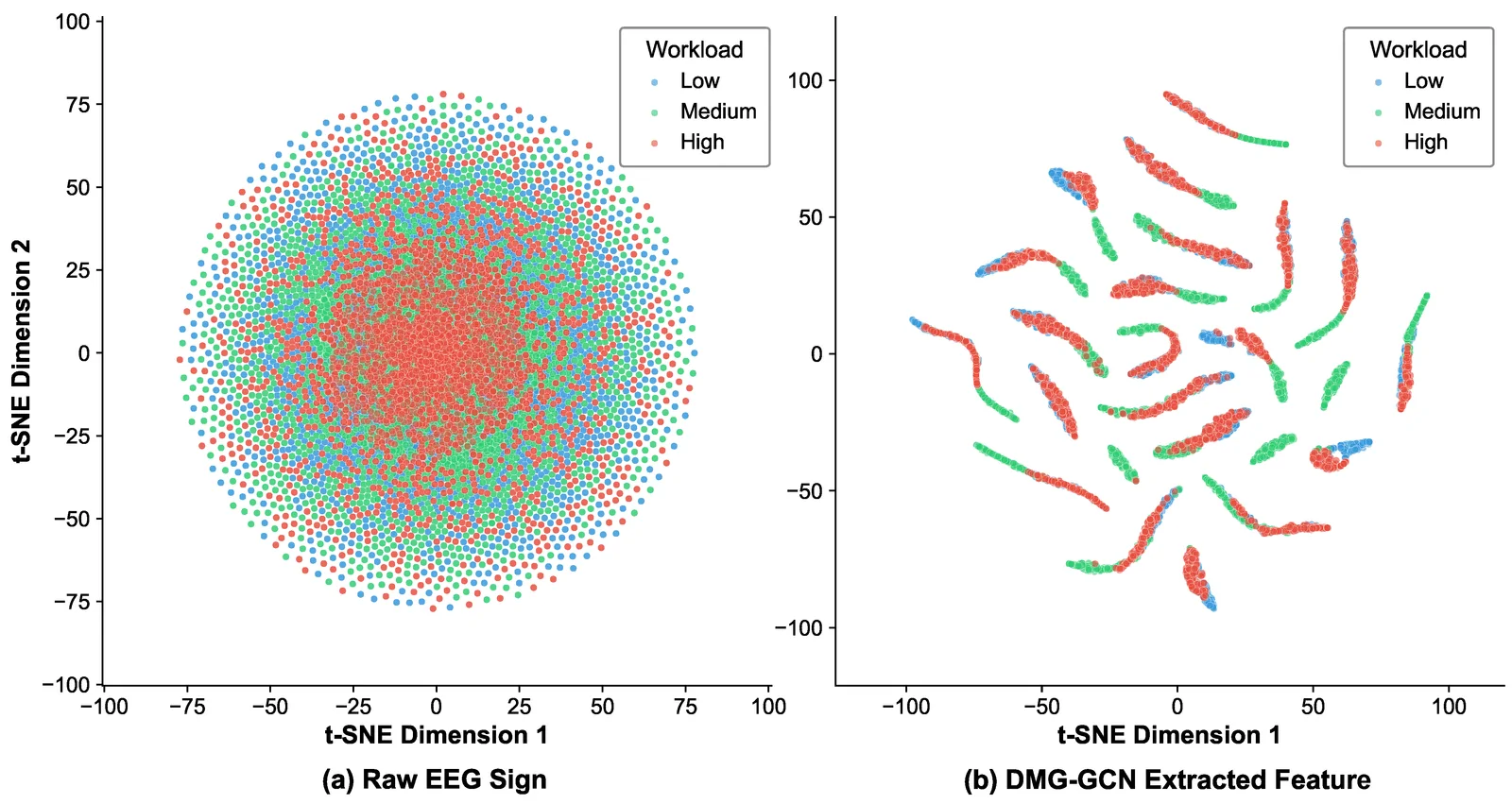
Two-dimensional t-SNE visualization comparing raw EEG signals and features extracted by DMG-GCN. Different colors represent distinct cognitive workload levels.

To intuitively evaluate the feature extraction and discriminative capabilities of the DMG-GCN model, this section employs the t-distributed Stochastic Neighbor Embedding (t-SNE) algorithm to project the high-dimensional EEG data into a two-dimensional space before and after model processing. Figure 8 presents a comparison of the feature distributions for all 21 subjects at the input stage (raw EEG signals) and the output stage (deep network features extracted by DMG-GCN). The blue, green, and red scatter points correspond to the low, medium, and high cognitive workload samples, respectively.

As shown in Figure 8a, within the feature space of raw EEG signals, the sample points of the three cognitive workload states exhibit a highly overlapping pattern, lacking clear clustering boundaries between different categories. This phenomenon reflects the inherent non-linear and non-stationary characteristics of scalp EEG signals, indicating severe feature overlapping among neural responses induced by distinct workloads in the original temporal domain. Such a disordered data distribution further substantiates the limitations of relying solely on shallow physical features for classification in air traffic control cognitive workload decoding tasks.

In contrast, as illustrated in Figure 8b, following the processing by the DMG-GCN model, the samples demonstrate a significant trend of intra-class aggregation and inter-class separation within the two-dimensional latent space. The feature distributions of the three workload states form three relatively independent clusters with distinct boundaries. Notably, the medium workload samples (green scatter points), which are severely confounded with other categories in Figure 8a, also obtain a well-defined and independent feature subspace in Figure 8b.

This comparative visualization corroborates the rationality and effectiveness of the proposed architecture from the perspective of feature representation. The results demonstrate that the multi-microstate topological disentanglement mechanism incorporated in DMG-GCN can effectively filter task-irrelevant redundant information from highly coupled raw EEG signals, thereby precisely extracting the underlying topological network patterns highly correlated with specific cognitive workloads. This capability to map complex non-linear signals into highly discriminative features provides an intuitive explanatory foundation for the superior classification performance reported in the preceding sections.

### 4.5. Mechanism Analysis of Dynamic Microstate Brain Network Topologies

**Figure 9:**
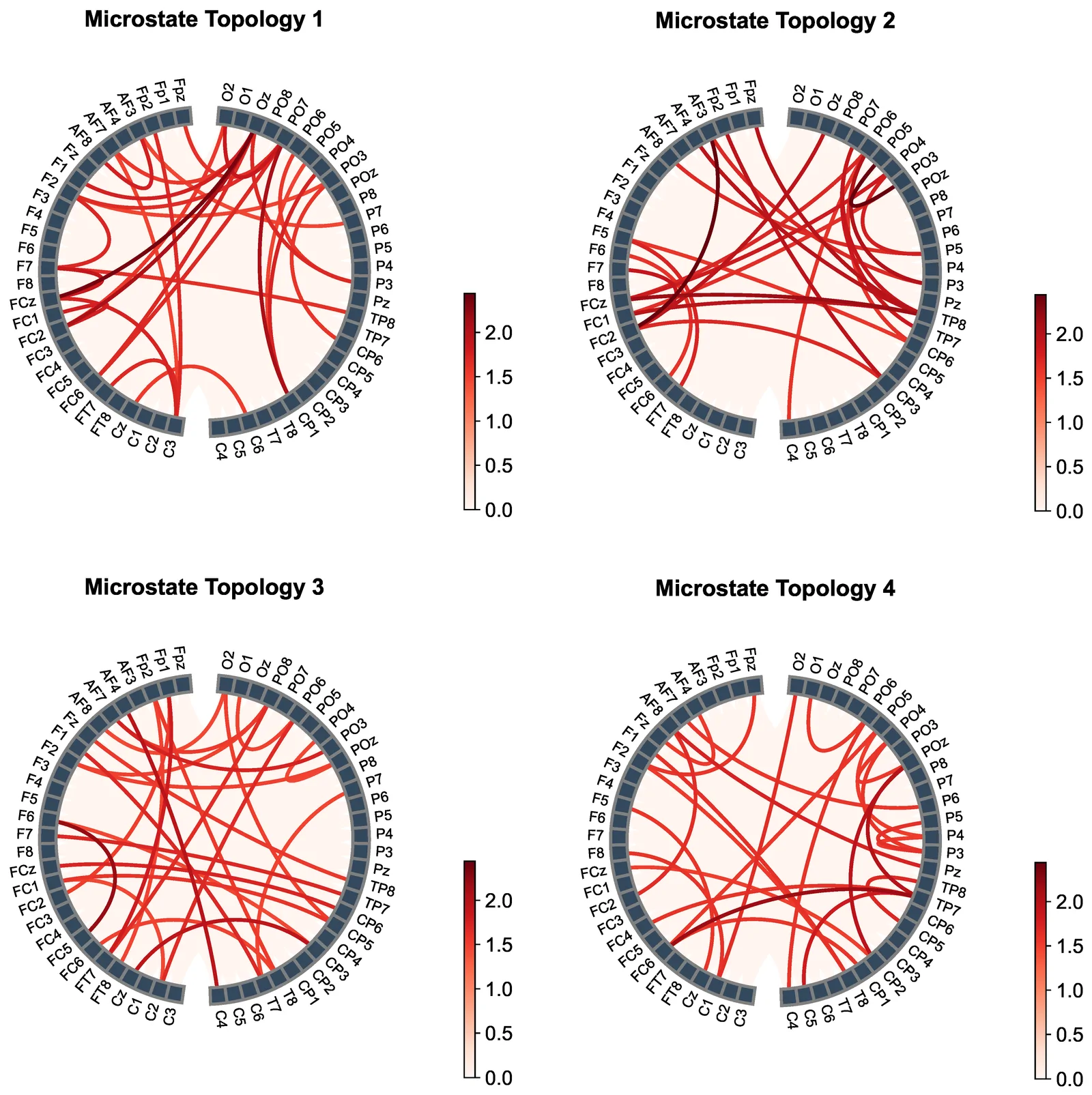
Four sets of microstate topological connectivity networks learned adaptively by the DMG-GCN model. The color depth and thickness of the edges represent the functional connection strength between nodes, displaying only the core topological paths ranking within the top 3% of connection strength.

To evaluate whether the features extracted by the DMG GCN model possess potential neurophysiological significance, this section performs an exploratory network visualization analysis on the connectivity basis matrices learned by the topology generation branch. This analysis aims to observe the data-driven reorganization patterns governing the internal functional connectivity modes of the brain when adapting to varying cognitive workloads. Figure 9 illustrates the four sets of spatial microstate topologies learned autonomously by the model. To ensure a focused analysis, only the core topological edges ranking within the top 3% of the connection strengths in each basis matrix are displayed.

As shown in Figure 9, the four learned topologies exhibit distinct spatial distribution patterns. This observation suggests that the model is capable of dividing complex network signals into multiple functional subnetworks characterized by unique connectivity configurations. While these representations are primarily data-driven and optimized for task-specific decoding, their spatial regional activations exhibit exploratory patterns that suggest a potential correlation with established cognitive networks. Combined with the spatial brain region mapping of the electrodes, specific connectivity patterns can be tentatively identified:

InMicrostate Topology 1, the network presents a prominentParietal–Occipital Coupling and localized clustering configuration. High-intensity connectivity paths primarily exist between the parietal and occipital lobes (e.g., the FCz–Oz path with a strength of 2.36, and the CP1–PO7 path with a strength of 2.09), accompanied by local interconnections within the frontal pole area (FPA) (e.g., AF3–AF8 with a strength of 1.77). In cognitive neuroscience, the synchronization of parietal–occipital networks is typically associated with baseline visual–spatial processing and sustained attention. This topological structure reflects the neural activation state of the participants maintaining routine radar screen scanning and visual information processing during low workload or baseline task phases.

InMicrostate Topology 2, the network configuration shifts into aHub-convergence structure centered on the right temporal region (RTemp). Electrode TP8 emerges as a prominent hub node, centrally receiving multiple long-range inputs from the frontal pole area (Fpz–TP8 with a strength of 1.99; AF4–TP8 with a strength of 1.96) and the parietal region (FCz–TP8 with a strength of 2.17; FC1–TP8 with a strength of 1.98). This high-intensity convergence of information from the frontal and parietal lobes toward the temporal lobe represents a cross-regional integration process of multi-modal information, such as visual–spatial telemetry and auditory/air-ground voice communications.

In contrast, Microstate Topology 3 manifests as a moreDistributed Cross-Regional Coordination. Its core edges are scattered across the whole brain, including cross-hemispherical connections between the right and left ventrolateral prefrontal cortices (F6–FC5 with a strength of 2.19), long-range paths from the frontal pole to the parietal lobe (AF4–C4 with a strength of 1.97), and connectivity from the dorsolateral prefrontal cortex (DLPFC) to the left temporal region (F1–T7 with a strength of 1.70). This loose, multi-centric parallel topology maps the dynamic transition and allocation of specific cognitive resources among different cortical zones. This topological switching mechanism provides neuro-computational evidence supporting the conclusion in the preceding ablation study that medium-workload decoding relies heavily on the dynamic multi-graph architecture.

InMicrostate Topology 4, the network evolves into a global complex framework characterized byPrefrontal–Temporal–Occipital Integration. Its strong connectivity paths involve distant coupling from the right ventrolateral prefrontal cortex to the left temporal region (FC6–TP7 with a strength of 2.17) and subsequent connectivity from the left temporal lobe to the occipital lobe (TP7–POz with a strength of 1.99). Under radar control scenarios of exceptionally high cognitive workloads, the participants must simultaneously execute high-frequency voice communications (temporal lobe), intensive visual searching (occipital lobe), and complex conflict resolution decision-making (prefrontal cortex). This microstate adaptively extracts this cross-modal, high-density synergetic sub-network.

It is mathematically crucial to clarify that the structural consistency of these four basis topologies is guaranteed across various subjects. Because the spatial prototype vectors are instantiated as global learnable parameters optimized simultaneously across the entire training corpus, the resulting connectivity matrices represent unified cross-subject functional approximations. The individual and task-specific physiological variations are dynamically captured exclusively through the real-time activation probabilities, ensuring that the foundational topological structures remain strictly consistent across all participants. In summary, the visualization analysis of the connectivity basis matrices suggests that the microstate disentanglement module employed in DMG-GCN can explore potential network reorganization processes consistent with actual human cognitive dynamics. While guaranteeing high classification accuracy, the model architecture offers exploratory neural interpretability, establishing a theoretical foundation for its deployment in civil aviation intelligent decision support systems.

### 4.6. Analysis of Dynamic Activation Distributions and Significance for Microstate Topologies

**Figure 10:**
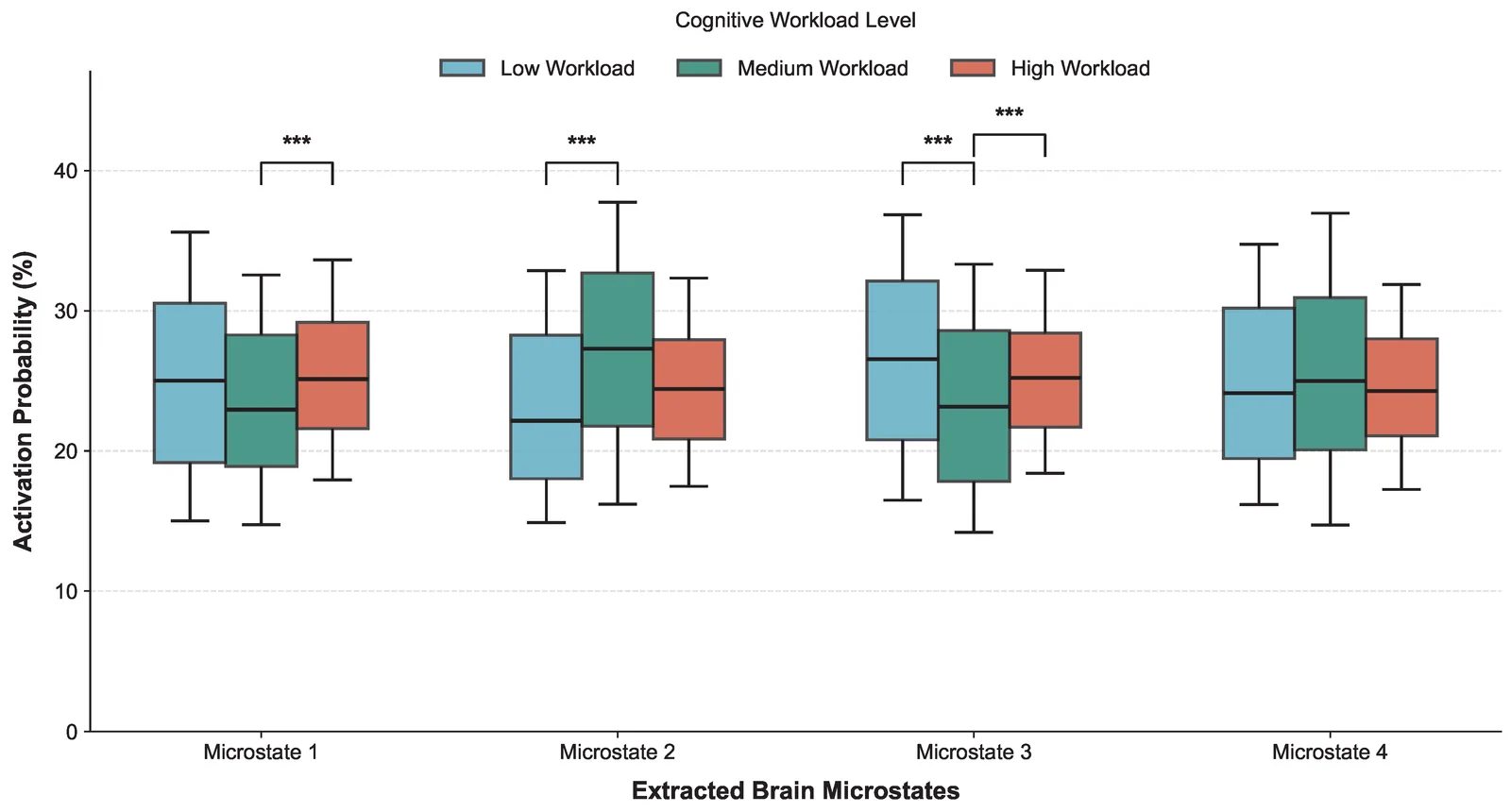
Dynamic activation probability distributions of the four microstate topologies under different cognitive workload conditions. The box plots illustrate the data distribution intervals of the samples, and the asterisks (***) denote highly significant statistical differences (p<0.001) in activation probabilities between distinct workload conditions.

Previous analyses have suggested that the DMG-GCN model is capable of decoupling microstate topological networks with specific physiological significance. To investigate the dynamic scheduling mechanisms of these functional sub-networks under varying task difficulties, this section performs a one-way repeated measures analysis of variance on the microstate activation probabilities generated by the model to properly account for the within-subject design. Subsequent pairwise comparisons were strictly evaluated using Bonferroni-corrected post hoc tests. The dynamic activation probability distributions and statistical significance test results for all subjects across low, medium, and high cognitive workloads are presented in Figure 10.

As indicated by the statistical test results in Figure 10, the activation weights of the microstate networks exhibit statistically significant adaptive modulation in response to changes in cognitive workload. Combined with the brain region topological features discussed previously, the specific network scheduling patterns are analyzed as follows:

Under thelow-workload condition, the activation level of Microstate 3 demonstrates a significant dominance with a mean activation probability of 26.63%. The repeated measures analysis reveals a significant main effect of workload levels on Microstate 3 activation ($F\left(2,40\right)=14.85,p<0.001,\eta_{p}^{2}=0.426$). Post hoc comparisons confirm that its activation intensity is significantly greater than that in the medium-workload phase (23.56%, p<0.001). Microstate 3 corresponds to a whole-brain distributed network. In cognitive neuroscience, such large-scale and low density functional connectivity is typically associated with general vigilance and environmental information monitoring. This phenomenon suggests that during low-density flight monitoring phases, subjects primarily rely on the discrete distribution of whole brain networks to maintain baseline sustained attention.

As task difficulty escalates to amedium workload, the activation probability of Microstate 2 exhibits a highly significant increase reaching 27.21%. Statistical evaluations demonstrate a strong main effect for this topology ($F\left(2,40\right)=16.32,p<0.001,\eta_{p}^{2}=0.449$). Bonferroni-corrected comparisons reveal this activation is significantly higher than both the low-workload (23.22%, p<0.001) and high-workload (24.65%, p<0.001) phases. Microstate 2 is characterized by a convergent topology centered on the right temporal lobe. The temporal lobe is a critical brain region for processing auditory information and multimodal information integration. The increased frequency of air–ground communications and preliminary spatial conflicts associated with medium workloads compel the brain to alter its baseline distributed synergetic strategy by enhancing information input from the frontal and parietal lobes to the temporal lobe, thereby accomplishing the cross-modal integration of auditory instructions and visual situations.

Under thehigh-workload condition, the network activation mechanism evolves into parallel network processing. Statistical data indicate a significant main effect for Microstate 1 ($F\left(2,40\right)=12.76,p<0.001,\eta_{p}^{2}=0.389$). The activation probability of Microstate 1 increases significantly during this phase rising from 23.51% in the medium-workload phase to 25.47% (p<0.001), maintaining an equivalently high level of activity alongside Microstate 3 (25.51%, p<0.001). Microstate 1 corresponds to the parietal-occipital visual network, which plays a core role in advanced visual-spatial attention and working memory tasks. In extreme high-workload scenarios, multi-aircraft conflict resolution requires the trainees to perform high-frequency visual searches and spatial trajectory predictions while simultaneously managing the scheduling of global situational resources. The significant increase in the relevant statistical data objectively corroborates the mandatory activation of specific visual spatial networks by the brain under high-pressure task states.

In summary, the statistical test results verify that the microstates extracted by DMG GCN possess a high degree of cognitive state specificity. This dynamic activation distribution objectively maps the neural mechanism reorganization process within the brains of the participants, transitioning from whole brain distributed monitoring under low workloads to multimodal information integration under medium workloads and ultimately evolving into parallel network processing under high workloads.

### 4.7. Computational Complexity and Real-Time Feasibility Analysis

To verify whether the proposed framework satisfies the stringent low-latency requirements for practical brain–computer interface deployments in adaptive automation, we conducted a comprehensive evaluation of its computational complexity and inference speed. We assessed three critical metrics: Parameter Count, Floating Point Operations (FLOPs), and Average End-to-End Inference Time.

The evaluation reveals that the proposed DMG-GCN framework comprises approximately 1.24 million learnable parameters and requires 485.6 million FLOPs to process a single input tensor. While the integration of the dynamic microstate topology generator and multi-scale temporal convolutions introduces additional computational overhead compared to shallow linear classifiers, this scale remains highly efficient and is easily manageable for modern standard computing hardware without necessitating high-performance clusters.

Crucially, the primary metric dictating the viability of continuous cognitive monitoring is the end-to-end inference latency. To ensure a realistic evaluation, the inference time was measured by incorporating the entire pipeline overhead, including data window slicing, band-pass filtering preprocessing, and CPU-to-GPU data transfer. Tested across 1000 independent iterations on an NVIDIA GeForce RTX 4060 GPU, the average end-to-end processing time for a single 3-s EEG data window was recorded at 118.5 ms. According to the data representation protocol detailed in Section 3.1, the continuous signals are evaluated using a sliding window step of Δ=1 s (1000 ms). Because the total inference latency (118.5 ms) is significantly shorter than the data generation interval (1000 ms), the model can effortlessly process incoming data streams without causing temporal accumulation or computational bottleneck. This quantitative analysis confirms that the DMG-GCN framework strictly satisfies the real-time processing constraints, validating its practical suitability for continuous cognitive workload monitoring in dynamic air traffic control environments.

## 5. Discussion

### 5.1. Methodological Contributions and Neurophysiological Insights

While electroencephalography offers exceptional temporal resolution for capturing instantaneous cognitive fluctuations, decoding cognitive workload in realistic environments is severely hindered by complex inter-subject variability and operational scenario shifts. Existing decoding approaches typically map multifaceted cognitive demands into homogenous neural representations or rely on static graph structures, which fail to disentangle the highly confounded physiological features caused by concurrent tasks. Furthermore, while recent dynamic graph neural networks attempt to address temporal non-stationarity, their reliance on unconstrained mathematical optimizations often produces opaque network topologies that lack domain-specific physiological validity. To address this fundamental methodological limitation, we propose the DMG-GCN framework, which establishes a dynamic topological disentanglement strategy driven by physiological constraints. Specifically, the dynamic selective kernel temporal convolutional block adaptively extracts multi-scale temporal–spectral dynamics, while the microstate-guided dynamic graph block strictly embeds microstate theory as a physiological prior. This architecture forces the autonomous learning of distinct connectivity basis matrices to align with genuine neural reorganizations, representing a fundamental departure from purely statistical graph generation. By explicitly isolating task-specific functional sub-networks from heavily coupled background noise, the proposed framework overcomes the feature confounding inherent in conventional mathematical models.

Ablation experiments confirm the necessity of each architectural component. The severe performance degradation observed in the Base GCN variant indicates that conventional static graph structures completely fail to capture the rapidly shifting functional brain network connectivity inherent in tactical ATC tasks. Furthermore, the significant performance gap between the variant without the microstate generator and the complete DMG-GCN framework, particularly under the highly dynamic medium-workload condition, demonstrates that the multi-microstate topological disentanglement mechanism is the key factor in effectively isolating overlapping neural representations. Comparisons with other advanced baselines further suggest that our proposed strategy yields superior results in terms of both decoding accuracy and cross-subject stability.

Crucially, the statistical validity of this functional network reorganization is rigorously substantiated by the repeated measures analysis of variance detailed in Section 4.6, which confirms consistent and highly significant activation shifts across all twenty-one subjects. Although these data-driven spatial patterns demonstrate a tentative alignment with established cognitive networks, such as temporal multimodal integration and parietal–occipital parallel processing, they inherently represent task-optimized functional approximations rather than definitive anatomical connectivity proofs.

Furthermore, it is essential to situate the proposed architecture within the broader theoretical context of multimodal data integration. The spatiotemporal dynamic graph convolutional fusion layer utilized in this study operates fundamentally as an early fusion strategy, comprehensively integrating temporal–spectral dynamics and spatial topological structures at the feature representation level. As thoroughly analyzed in [37], early fusion theoretically possesses an optimal classification capacity compared to late fusion when the data model is adequately captured, which mathematically substantiates the superior decoding accuracy achieved by our DMG-GCN framework over the individual baseline methods. However, this literature also mathematically demonstrates that early fusion paradigms are highly susceptible to dimensionality challenges and necessitate exponentially larger training sets to maintain stability. Given the inherent constraints of collecting extensive calibration data in real-world air traffic control brain–computer interfaces, exploring a late fusion strategy represents a highly critical direction for future operational deployments. Specifically, implementing a decision-level late fusion ensemble that aggregates the independent predictive scores of the diverse baseline models evaluated in our current study could effectively mitigate the dimensionality constraints of early fusion. This strategic transition from feature-level to decision-level integration offers a promising pathway to further enhance the robustness of cognitive workload decoding under strict data limitations.

### 5.2. Limitations of the Proposed Study

Despite the encouraging decoding performance and exploratory neurophysiological insights achieved by the proposed framework, several limitations warrant explicit acknowledgment. First, the experimental paradigm relies on a simulated aerodrome control environment. Although this design facilitates precise modulation of cognitive workload gradients, it inherently lacks the multifaceted operational stressors and severe consequences present in actual air traffic operations. Consequently, the elicited neurophysiological responses may not fully encapsulate the complex cognitive dynamics of real operational scenarios.

Second, the participant cohort in this study consists of specialized trainees rather than fully certified air traffic controllers, and the evaluations were restricted to internal cross-validation protocols. Professional controllers possess distinct cognitive resource allocation strategies and neural efficiency developed through long-term operational experience. Currently, due to stringent aviation safety regulations and the practical constraints of deploying high-density EEG equipment in active control centers, collecting real-world physiological data from professionals on duty remains highly challenging. This demographic discrepancy, coupled with the lack of external validation on independent datasets, restricts the immediate generalizability of the trained model to professional populations. Therefore, the current framework serves as a rigorous proof-of-concept. To rigorously verify the robustness and broader applicability of the dynamic topological disentanglement strategy, future investigations must prioritize validating the framework against external operational datasets collected from professional cohorts, eventually bridging the gap toward real-world deployment.

Furthermore, the experimental design introduces potential confounding effects regarding sensory and task content variations across the defined workload levels. Specifically, providing visual text support in the low-workload condition and introducing an explicit mental arithmetic task in the high-workload condition inevitably alter the sensory modalities and physical responses required from the participants. While this progressive design maximizes ecological validity by accurately mirroring the multifaceted escalation of multimodal demands in actual air traffic operations, it inherently embeds sensory and motor confounds into the recorded electroencephalography signals. Consequently, the proposed framework might be partially decoding these hybrid sensory motor shifts alongside the intrinsic cognitive workload. Future experimental paradigms must consider isolating purely cognitive variations while strictly controlling for visual and auditory stimulus uniformity to systematically decouple these confounding factors.

Finally, regarding the methodological mechanisms, the current interpretations of the learned graph structures remain largely qualitative. Because these dynamic topologies are optimized for cognitive workload decoding rather than quantitatively evaluated against canonical electroencephalography microstate templates or structural neuroimaging, conclusions regarding their direct equivalence to classical functional brain networks should be approached with rigorous caution. Future work will focus on expanding the cross-subject database in realistic simulators and exploring fully adaptive prototype generation algorithms to further enhance the operational robustness of the model.

## 6. Conclusions

In this paper, we model the multi-scale temporal–spectral dynamics and dynamic spatial topological features of EEG signals and propose a novel framework named DMG-GCN. Specifically, the DSK-TCB module adaptively extracts multi-scale local temporal–spectral features from individual nodes, and the MG-DGB module constructs a time-evolving graph adjacency matrix guided by EEG microstate priors. Furthermore, the spatiotemporal dynamic graph convolution module enables deep interactive fusion to integrate local spectral features with global functional connectivity structures. Ablation experiments evaluate the contribution of each module. On the simulated aerodrome control dataset, the proposed model achieves an overall average accuracy of 80.30%, suggesting that the proposed dynamic topological disentanglement strategy isolates overlapping neural representations and provides a foundational framework for exploring air traffic controller cognitive workload decoding in future operational applications.

## Figures and Tables

**Table 1:** Brain Regions and their Corresponding Electrodes.

Brain Region	Electrode Name
Frontal Pole Area (FPA)	Fpz, Fp1, Fp2, AF3, AF4, AF7, AF8
Dorsolateral Prefrontal Cortex (DLPFC)	Fz, F1, F2, F3, F4
Parietal	FCz, FC1, FC2, FC3, FC4, Cz, C1, C2, C3, C4, CP1, CP2, CP3, CP4
Occipital	Pz, P3, P4, POz, PO3, PO4, PO5, PO6, PO7, PO8, Oz, O1, O2
Left Ventrolateral Prefrontal Cortex (LVLPFC)	F5, F7, FC5, FT7
Right Ventrolateral Prefrontal Cortex (RLPFC)	F6, F8, FC6, FT8
Left Temporal (LTemp)	C5, CP5, P5, T7, TP7, P7
Right Temporal (RTemp)	C6, CP6, P6, T8, TP8, P8

**Table 2:** Performance Comparison of Advanced Baseline Models and the Proposed Method Across Different Cognitive Workloads (Mean ± Std %). The bold values indicate the best performance in each evaluation metric.

Model	Metrics	Low Workload	Medium Workload	High Workload	Overall Average
SVM	Accuracy (%)	58.27 ± 9.41	50.84 ± 10.38	59.19 ± 9.67	56.10 ± 8.79
	F1-Score (%)	56.03 ± 10.11	48.16 ± 11.23	56.78 ± 10.42	53.66 ± 9.44
	Recall (%)	57.11 ± 9.88	49.52 ± 10.74	58.05 ± 10.15	54.89 ± 9.17
EEGNet	Accuracy (%)	67.81 ± 7.63	60.36 ± 8.49	66.92 ± 7.84	65.03 ± 7.15
	F1-Score (%)	66.14 ± 8.12	58.07 ± 9.14	64.83 ± 8.37	63.01 ± 7.62
	Recall (%)	66.95 ± 7.78	59.21 ± 8.76	65.74 ± 8.02	63.97 ± 7.30
EEG-Conformer	Accuracy (%)	72.94 ± 6.17	66.15 ± 7.31	72.48 ± 6.62	70.52 ± 5.95
	F1-Score (%)	71.38 ± 6.74	63.82 ± 8.06	70.61 ± 7.23	68.60 ± 6.49
	Recall (%)	72.13 ± 6.45	64.91 ± 7.62	71.55 ± 6.88	69.53 ± 6.22
ASTGCN	Accuracy (%)	76.38 ± 5.46	69.57 ± 6.13	74.61 ± 5.79	73.52 ± 5.21
	F1-Score (%)	74.82 ± 6.04	67.63 ± 6.91	73.19 ± 6.38	71.88 ± 5.72
	Recall (%)	75.56 ± 5.72	68.49 ± 6.47	73.94 ± 6.11	72.66 ± 5.44
LMDA-Net	Accuracy (%)	80.05 ± 4.42	72.18 ± 5.57	77.83 ± 4.71	76.69 ± 4.34
	F1-Score (%)	78.61 ± 4.98	70.06 ± 6.24	76.27 ± 5.31	74.98 ± 4.88
	Recall (%)	79.37 ± 4.65	71.13 ± 5.86	77.08 ± 4.96	75.86 ± 4.56
**DMG-GCN (Ours)**	Accuracy (%)	**84.13 ± 3.82**	**75.47 ± 4.56**	**81.29 ± 4.08**	**80.30 ± 4.05**
	F1-Score (%)	**82.76 ± 4.11**	**73.18 ± 5.03**	**79.94 ± 4.35**	**78.63 ± 3.72**
	Recall (%)	**83.05 ± 3.96**	**74.02 ± 4.78**	**80.53 ± 4.21**	**79.20 ± 4.04**

**Table 3:** Performance Comparison of Model Variants Across Different Cognitive Workloads (Mean ± Std %).

Model	Metrics	Low Workload	Medium Workload	High Workload	Overall Average
Base GCN	Accuracy (%)	48.65 ± 10.37	41.28 ± 12.05	46.92 ± 11.23	45.62 ± 11.00
	F1-Score (%)	46.21 ± 11.08	38.64 ± 13.25	44.38 ± 12.14	43.08 ± 10.17
	Recall (%)	47.19 ± 10.76	39.75 ± 12.83	45.47 ± 11.62	44.14 ± 10.94
w/o Microstate	Accuracy (%)	67.84 ± 6.71	59.32 ± 8.14	65.61 ± 7.45	64.26 ± 7.26
	F1-Score (%)	66.12 ± 7.24	56.87 ± 8.86	63.79 ± 7.91	62.26 ± 7.02
	Recall (%)	66.98 ± 6.95	58.11 ± 8.52	64.55 ± 7.63	63.21 ± 7.22
w/o Temporal Conv	Accuracy (%)	76.25 ± 5.12	67.68 ± 6.35	73.42 ± 5.88	72.45 ± 5.78
	F1-Score (%)	74.85 ± 5.60	65.23 ± 7.10	71.98 ± 6.45	70.69 ± 6.38
	Recall (%)	75.42 ± 5.45	66.15 ± 6.80	72.65 ± 6.20	71.41 ± 6.15
w/o Attention	Accuracy (%)	80.12 ± 4.45	71.35 ± 5.25	77.07 ± 4.86	76.18 ± 4.85
	F1-Score (%)	78.65 ± 4.80	69.25 ± 5.85	75.76 ± 5.30	74.55 ± 5.31
	Recall (%)	79.20 ± 4.65	70.18 ± 5.60	76.45 ± 5.15	75.28 ± 5.13
w/o Label Smoothing	Accuracy (%)	82.35 ± 4.10	73.68 ± 4.85	79.59 ± 4.35	78.54 ± 4.43
	F1-Score (%)	80.95 ± 4.40	71.45 ± 5.30	78.22 ± 4.65	76.87 ± 4.78
	Recall (%)	81.52 ± 4.25	72.35 ± 5.10	78.85 ± 4.50	77.57 ± 4.61
**DMG-GCN (Ours)**	Accuracy (%)	**84.13 ± 3.82**	**75.47 ± 4.56**	**81.29 ± 4.08**	**80.30 ± 4.05**
	F1-Score (%)	**82.76 ± 4.11**	**73.18 ± 5.03**	**79.94 ± 4.35**	**78.63 ± 3.72**
	Recall (%)	**83.05 ± 3.96**	**74.02 ± 4.78**	**80.53 ± 4.21**	**79.20 ± 4.04**

## Data Availability

The data presented in this study are available on request from the corresponding author due to privacy and ethical restrictions.

## Abbreviations

The following abbreviations are used in this manuscript:

ATCO	Air Traffic Controller
EEG	Electroencephalography
CW	Cognitive Workload
BCI	Brain–Computer Interface
DMG-GCN	Dynamic Microstate-Guided Graph Convolutional Network
DSK-TCB	Dynamic Selective Kernel Temporal Convolutional Block
MG-DGB	Microstate-Guided Dynamic Graph Block
PSD	Power Spectral Density
FPA	Frontal Pole Area
DLPFC	Dorsolateral Prefrontal Cortex
